# Global trends and hotspots of ChatGPT in medical research: a bibliometric and visualized study

**DOI:** 10.3389/fmed.2024.1406842

**Published:** 2024-05-16

**Authors:** Ling Liu, Shenhong Qu, Haiyun Zhao, Lingping Kong, Zhuzhu Xie, Zhichao Jiang, Pan Zou

**Affiliations:** ^1^Nanxishan Hospital of Guangxi Zhuang Autonomous Region (The Second People’s Hospital of Guangxi Zhuang Autonomous Region), Guilin, China; ^2^School of Integrated Chinese and Western Medicine, Hunan University of Chinese Medicine, Changsha, China; ^3^Department of Otolaryngology-Head and Neck Oncology, The People’s Hospital of Guangxi Zhuang Autonoms Region, Nanning, China; ^4^Hunan Provincial Brain Hospital, The Second People’s Hospital of Hunan Province, Changsha, China

**Keywords:** global trends, ChatGPT, medical research, bibliometric, visualized

## Abstract

**Objective:**

With the rapid advancement of Chat Generative Pre-Trained Transformer (ChatGPT) in medical research, our study aimed to identify global trends and focal points in this domain.

**Method:**

All publications on ChatGPT in medical research were retrieved from the Web of Science Core Collection (WoSCC) by Clarivate Analytics from January 1, 2023, to January 31, 2024. The research trends and focal points were visualized and analyzed using VOSviewer and CiteSpace.

**Results:**

A total of 1,239 publications were collected and analyzed. The USA contributed the largest number of publications (458, 37.145%) with the highest total citation frequencies (2,461) and the largest *H*-index. Harvard University contributed the highest number of publications (33) among all full-time institutions. The *Cureus Journal of Medical Science* published the most ChatGPT-related research (127, 10.30%). Additionally, Wiwanitkit V contributed the majority of publications in this field (20). “Artificial Intelligence (AI) and Machine Learning (ML),” “Education and Training,” “Healthcare Applications,” and “Data Analysis and Technology” emerged as the primary clusters of keywords. These areas are predicted to remain hotspots in future research in this field.

**Conclusion:**

Overall, this study signifies the interdisciplinary nature of ChatGPT research in medicine, encompassing AI and ML technologies, education and training initiatives, diverse healthcare applications, and data analysis and technology advancements. These areas are expected to remain at the forefront of future research, driving continued innovation and progress in the field of ChatGPT in medical research.

## 1 Introduction

Chat Generative Pre-Trained Transformer (ChatGPT), developed by OpenAI (San Francisco, CA, United States), is an innovative artificial intelligence language model revered for its remarkable natural language processing capabilities (1). It excels in engaging conversations, delivering information, crafting text, and mirroring human speech patterns. Its versatility spans aiding users, enriching educational experiences, generating contents, and facilitating research endeavors (2). Fueled by extensive knowledge and adaptive learning, ChatGPT continually evolves, pushing the boundaries of AI technology. Its ability to understand context, infer meaning, and produce coherent responses has garnered widespread acclaim, making it a pivotal tool across various industries. From customer service chatbots to personalized content generation and even creative writing assistance, ChatGPT’s impact is far-reaching. As it continues to evolve and improve, ChatGPT holds immense promise for revolutionizing human-computer interactions and advancing the capabilities of AI-powered solutions (3, 4).

The development history of ChatGPT spans several versions, each representing significant advancements in natural language processing technology. The journey began with GPT, or “Generative Pre-trained Transformer,” which laid the foundation for subsequent iterations. GPT-2, released in 2019, garnered attention for its impressive ability to generate coherent and contextually relevant text across diverse topics (5). Following this, OpenAI introduced GPT-3 in June 2020, notable for its unprecedented scale with 175 billion parameters, enabling more nuanced responses and improved context understanding (6). Building upon this success, OpenAI launched ChatGPT, a specialized variant fine-tuned for conversational interactions, in response to the growing demand for AI models tailored specifically for dialogue-based applications. In January 2022, ChatGPT has seen further refinements and optimizations, continually enhancing its conversational abilities and expanding its range of applications in various domains, including customer service, education, and healthcare (7, 8). Since its debut in November 2022, ChatGPT-3.5, equipped with transformer architecture capabilities, has attracted significant attention as a tool to enhance efficiency in medical practice and expedite medical research (9). The debut of ChatGPT-4 in February 2023 marked a significant advancement in ChatGPT’s functionality (10, 11). This version showcased notable improvements in accuracy, evidenced by higher ChatGPT test scores on standardized exams and a reduction in the dissemination of inaccurate information. Moreover, ChatGPT-4 introduced the capability to process inputs beyond text, including images and data. This expanded functionality enables ChatGPT to generate text based on a broader range of inputs, which holds promising implications for scientific research, particularly in manuscript writing (12).

Currently, ChatGPT is being utilized in various capacities within medical research, showcasing its versatility and potential impact in advancing healthcare (1). One prominent application is in natural language processing (NLP) tasks, where ChatGPT models are employed to analyze and generate text-based data such as medical records, clinical notes, and research literature (13). This facilitates tasks such as information extraction, summarization, and categorization, streamlining data interpretation and enabling researchers to extract valuable insights from large volumes of unstructured text data.

Moreover, ChatGPT models are increasingly utilized in virtual medical assistants and chatbots, providing personalized assistance to patients, caregivers, and healthcare professionals (14). These virtual assistants can offer support in scheduling appointments, providing medication reminders, answering medical inquiries, and even assisting in telemedicine consultations. By leveraging Chat GPT’s natural language understanding capabilities, these virtual assistants enhance patient engagement, improve healthcare accessibility, and alleviate the burden on healthcare providers.

In addition, ChatGPT plays a crucial role in medical education and training programs (15, 16). It serves as a valuable tool for simulating patient-doctor interactions, allowing medical students and professionals to practice clinical decision-making, communication skills, and diagnostic reasoning in a realistic virtual environment. Furthermore, ChatGPT-based educational platforms provide interactive learning experiences, delivering personalized feedback and adaptive learning pathways tailored to individual learners’ needs.

Furthermore, ChatGPT is employed in medical research for tasks such as literature review, hypothesis generation, and data synthesis (17). By analyzing vast repositories of scientific literature and clinical data, ChatGPT models can identify relevant research trends, discover novel associations, and generate hypotheses for further investigation. This accelerates the research process, facilitates knowledge discovery, and contributes to the advancement of medical science.

Bibliometrics, a quantitative method analyzing scholarly literature, assesses research impact through citation counts, publication frequencies, and authorship patterns, offering insights into academic publication influence and visibility (18–20). Commonly used across disciplines, bibliometrics, including citation counts, *H*-index, and journal impact factors, aids in understanding research trends, evaluating scholarly productivity, and informing decision-making processes in academia (21). Hence, it has been utilized to examine global trends in GPT-related research, such as plastic surgery (22), obstetrics and gynecology (23), and pediatric surgery (2). Barrington et al. (24) have conducted a bibliometric analysis of ChatGPT literature in medicine and science, aiming to gain deeper insights into publication trends and identify knowledge gaps in August 2023. Considering the exponential rise in articles discussing the application of ChatGPT in academia and medicine, conducting a bibliometric analysis becomes essential. In this study, we employ bibliometric tools such as CiteSpace and VOSviewer to visually illustrate trends in ChatGPT literature within healthcare domains over the past year. We also assess its utilization across different countries and specialties, identifying key features and anticipating future research trajectories.

## 2 Materials and methods

### 2.1 Data acquisition and search strategies

Publications on ChatGPT in medical research were retrieved using the Web of Science Core Collection (WoSCC) by Clarivate Analytics. Subsequently, studies related to ChatGPT in medical research were identified, and bibliometric and visualization analyses were conducted following established methodologies from previous studies. The search parameters were set from January 1, 2023, to January 31, 2024, with the search formula structured as follows: TS = (ChatGPT OR ChatGPT OR Chat-GPT) AND TS = (med* OR surg* OR physician OR doctor OR patient). Additionally, the publication criteria for inclusion are shown below: (1) publications primarily focused on ChatGPT in medical research (including medical research, medical education, and patient care), and (2) papers written in English. In addition, the exclusion criteria are: (1) publications are not focused on ChatGPT in medical research (including medical research, medical education, and patient care), and (2) papers not written in English. The publications underwent meticulous evaluation by relevant reviewers, and any publications not related to the topic of ChatGPT in medical research were manually filtered out. Furthermore, experienced corresponding authors were consulted to determine whether any potentially relevant but initially excluded publications should be included in this study.

The authors extracted basic information regarding the publications, including details such as journals, titles, authors, keywords, institutions, countries/regions, publication dates, as well as comprehensive statistics such as total citations, *H*-index, and average citation counts. This information was then imported into Excel 2021. Subsequently, bibliometric analyses and visualizations were conducted using a suite of software applications, including GraphPad Prism 8, Origin 2021, and VOSviewer (version 1.6.14, Leiden University, Leiden, The Netherlands) and CiteSpace (version 6.2.4). These tools play a pivotal role in dissecting and visualizing the intricate landscape of publications related to ChatGPT in medical research, offering a comprehensive perspective on the scholarly contributions in this domain.

### 2.2 Bibliometric analysis and visualization

Bibliometrics entails studying interconnected bodies of literature. It involves analyzing and visualizing links between research topics, researchers, affiliations, or journals. Initially, monthly publication trends and relative research interest (RRI) were graphically represented using the curve-fitting function in GraphPad Prism 8. RRI is calculated as the number of papers in a specific field divided by the total number of papers in all fields in a given month, offering insights into the prominence of the field relative to others. For the world map analysis, a methodology based on previous research was utilized (25). Furthermore, the total number of publications for the top 10 countries between January 1, 2023 and January 31, 2024 along with global trend projections, were analyzed using Origin 2021 software.

An in-depth examination of pertinent studies was undertaken utilizing VOSviewer and CiteSpace software (6.3.R1) tools to elucidate collaborations (co-authors), themes (terminology co-occurrence), and citation patterns (bibliographic coupling). This entailed scrutinizing country/region and institution collaborations, overlaying journal biplots, analyzing author collaborations, investigating co-cited authors, and performing cluster analyses. Furthermore, co-cited references and keywords were meticulously identified and assessed to highlight those with significantly higher citations. By configuring relevant parameters as described above, the analysis ensured robust and precise exploration of a substantial volume of literature data on ChatGPT in medical research.

Furthermore, this study employed VOSviewer to construct and visualize the bibliometric network, enabling a more comprehensive analysis that includes: (1) bibliographic coupling analysis of countries/regions, institutions, journals, and authors; (2) co-authorship analysis of countries/regions, institutions, and authors; (3) co-citation analysis of journals, references, and authors; and (4) keyword co-occurrence analysis. In the graphical representation generated by VOSviewer, each node corresponds to an entity containing co-cited references and keywords. The size of the node corresponds to the number of publications associated with it, while its color indicates the publication year. The thickness of the connecting lines between nodes reflects the strength of collaborative or co-citation relationships, providing an intuitive visual depiction of the complex interconnections within bibliographic data. This approach enhances the depth of understanding of thematic and conceptual associations within the realm of medical research involving ChatGPT in medical research.

## 3 Results

### 3.1 Global contribution to the field

**Figure 1:**
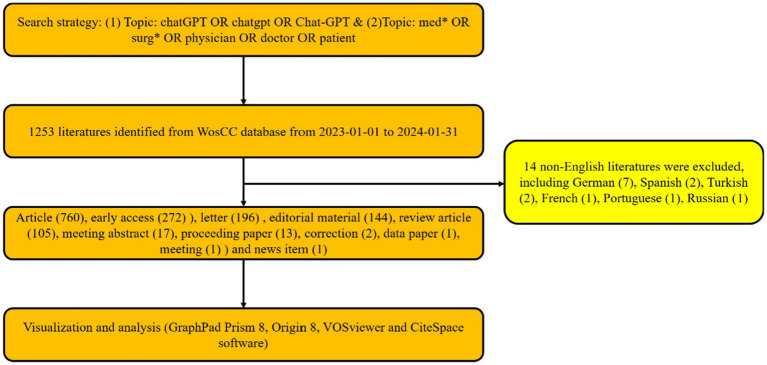
Flowchart depicting the literature selection process.

**Figure 2:**
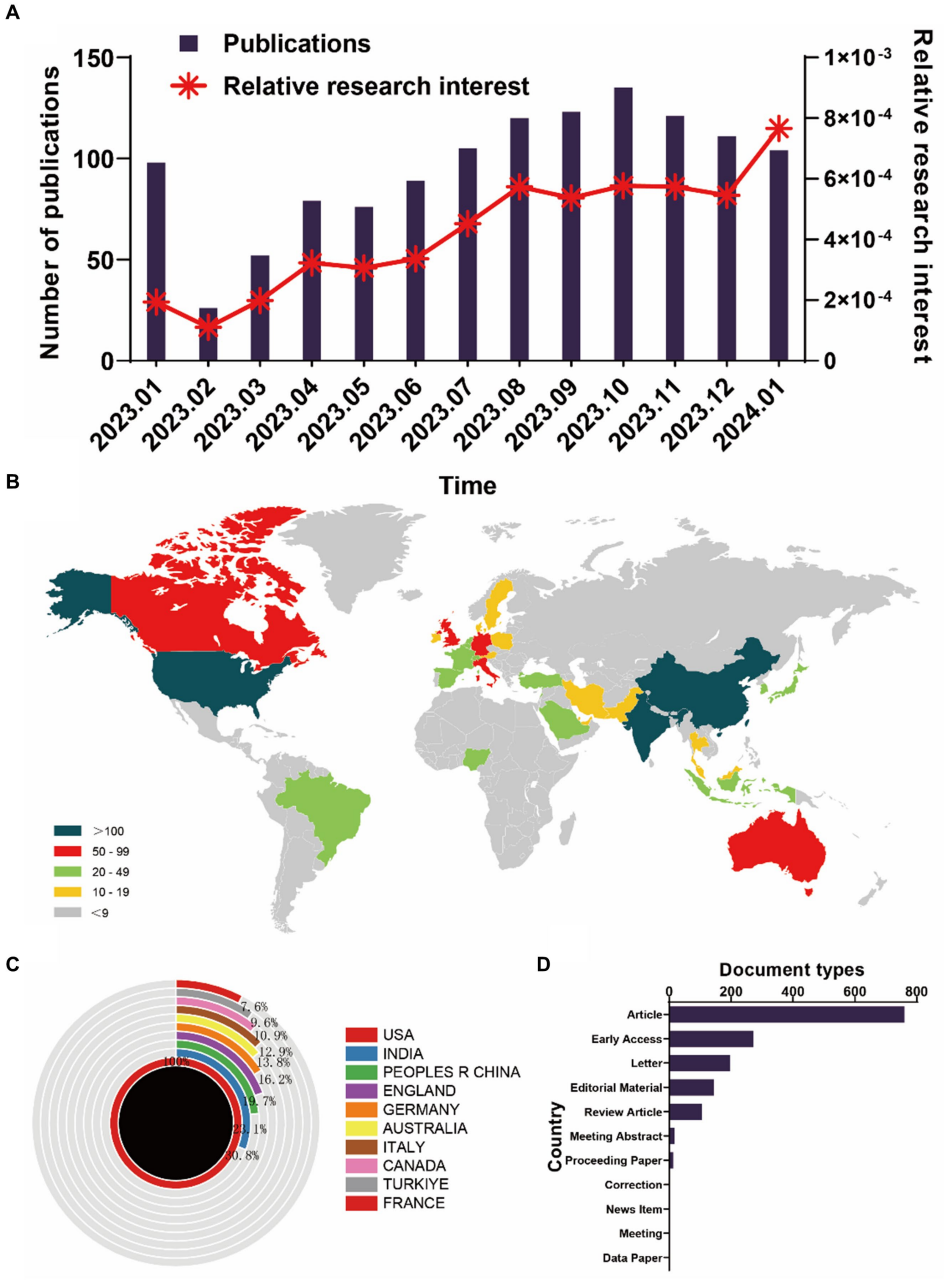
Global trends and the geographical landscape of ChatGPT in medical research. **(A)** Monthly publication statistics concerning ChatGPT in medical research. **(B)** A global map illustrating the dispersion of such research. **(C)** The cumulative publication counts across the top 10 most prolific countries. **(D)** The document types of all publications.

In the realm of global publications, a total of 1,239 articles from the WosCC database met the search criteria (Figure 1). From January 1, 2023, to January 31, 2024, the trajectory of global publications witnessed a steady monthly increase, escalating from a modest 26 articles (in February 2023) to an impressive 100+ articles (in January 2024, as depicted in Figure 2A). Simultaneously, the relative research interest (RRI) exhibited a relatively stable trend around the baseline level over the same period (as illustrated in Figure 2A). Overall, contributions in the domain of ChatGPT in medical research emanated from 91 different countries/regions. Notably, the USA spearheaded this effort with the largest share of 458 publications, comprising 37.145% of the total. This was succeeded by India (141 publications, 11.436%), China (106 publications, 8.597%), and England (90 publications, 7.299%) (depicted in Figures 2B,C). In recent years, several other countries have also made significant strides and remain competitive in advancing research endeavors. Furthermore, Figure 2D delineates the document types, with the top five categories being articles (760, 61.34%), early access (272, 21.9535%), letters (196, 15.819%), editorial materials (144, 11.622%), and review articles (105, 8.475%).

### 3.2 Quality of publications of different countries and regions

**Figure 3:**
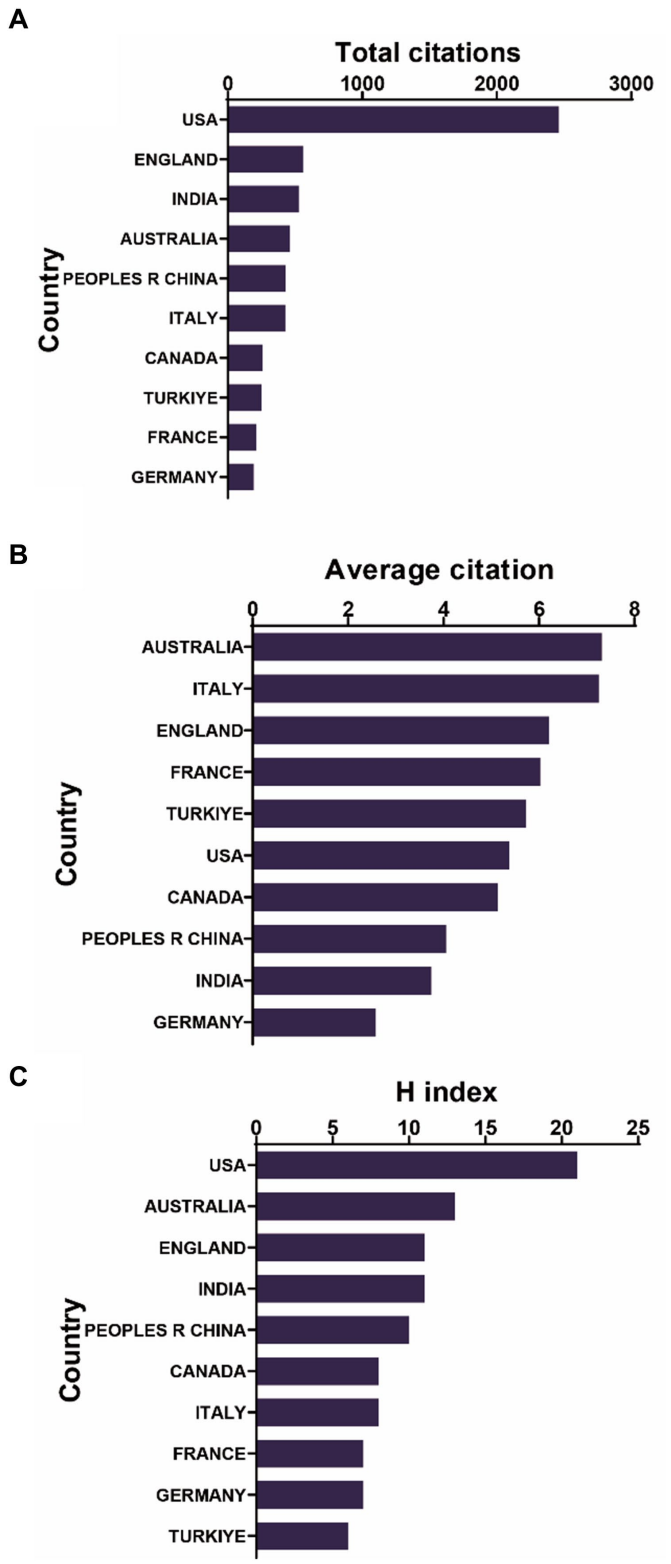
**(A)** The foremost 10 countries/regions boasting the highest cumulative citations in medical research focusing on ChatGPT. **(B)** The premier 10 countries/regions exhibiting the highest mean citations per publication in medical research revolving around ChatGPT. **(C)** The top-ranking 10 countries/regions with the most elevated publication *H*-index in medical research concerning ChatGPT.

In the analysis of total citation frequencies, publications originating from the USA garnered the highest total citation frequency (2,461). Following the USA, England ranked second with a total citation frequency of 559, trailed by India (528), Australia (461), and China (430) (Figure 3A). Moreover, publications from Australia recorded the highest average citation frequency (7.32). Italy followed closely in second place in terms of average citation frequency (7.25), succeeded by England (6.21), France (6.03), and Turkey (5.73) (Figure 3B). Additionally, regarding the relative publications, the USA led with the highest *H*-index (21), followed by Australia (13), England (11), India (11), and China (10) (Figure 3C).

### 3.3 Analysis of global leading institution output, funding sources, research orientation, authors and productive journals

Table 1 shows the top 10 institutions by publication volume, with eight institutions hailing from the USA. Harvard University (33), Stanford University (31), and the University of California System (31) consistently hold the top three positions. Only the University of London from the UK (22) and the National University of Singapore from Singapore (21) rank fifth and sixth, respectively.

**Table 1:** The top 10 institutions published literature related to ChatGPT in medical research.

Rank	Institution	Article counts	Percentage (*N*/1,239)
1	Harvard University	33	2.68
2	Stanford University	31	2.51
3	University of California System	31	2.51
4	Harvard Medical School	22	1.78
5	University of London	22	1.78
6	National University of Singapore	21	1.70
7	Duke University	20	1.62
8	Mayo Clinic	20	1.62
9	Pennsylvania Commonwealth System of Higher Education PCSHE	20	1.62
10	University of Pittsburgh	19	1.54

In addition, the top 10 funding sources are shown in Table 2. In total, 31 publications were funded by the National Institutes of Health (NIH) (ranked first) and the United States Department of Health and Human Services (ranked first), 23 publications were funded by the National Natural Science Foundation of China (NSFC) (ranked third), eight publications were funded by Projekt Deal and five publications were funded by China Postdoctoral Science Foundation. It is worth noting that seven articles did not receive any funding support.

**Table 2:** The top 10 funds related to ChatGPT in medical research.

Rank	Journal	Article counts	Percentage (*N*/1,239)
1	National Institutes of Health NIH USA	31	2.51
2	United States Department of Health and Human Services	31	2.51
3	National Natural Science Foundation of China NSFC	23	1.87
4	Projekt Deal	8	0.65
5	None	7	0.57
6	China Postdoctoral Science Foundation	5	0.41
7	NIH National Cancer Institute NCI	5	0.41
8	European Union EU	4	0.32
7	German Research Foundation DFG	4	0.32
10	Japan Society for the Promotion of Science	4	0.324

Furthermore, Table 3 provides a breakdown of the number of articles published in different research orientations, along with their respective percentages. “General Internal Medicine” has the highest number of articles, accounting for 18.25% of the total, followed by “Surgery” with 13.79%. Research orientations related to education, health care sciences, and engineering also contribute significantly, each comprising around 9% of the total articles. Other fields such as computer science, radiology, medical informatics, orthopedics, and oncology have varying but notable contributions ranging from 3 to 5%.

**Table 3:** Publication distribution by top 10 research domains.

Rank	Research areas	Article counts	Percentage (*N*/1,239)
1	General Internal Medicine	225	18.25
2	Surgery	170	13.79
3	Education Educational Research	113	9.17
4	Health Care Sciences Services	113	9.17
5	Engineering	71	5.76
6	Computer Science	65	5.27
7	Radiology Nuclear Medicine Medical Imaging	63	5.11
8	Medical Informatics	60	4.87
9	Orthopedics	41	3.33
10	Oncology	38	3.08

Table 4 presents the highly published authors along with the number of articles attributed to each author. Wiwanitkit V leads the list with 20 articles, followed by Kleebayoon A with 16 articles, and Seth I with 15 articles. Other authors such as Gupta R, Mondal H, Rozen WM, Wu HY, Cheungpasitporn W, Egro FM, and Klang E also contribute significantly, each having published between 8 to 10 articles.

**Table 4:** The top 10 authors with the most publications on ChatGPT in medical research.

Rank	Highly published authors	Article counts	Percentage (*N*/1,239)
1	Wiwanitkit V	20	1.62
2	Kleebayoon A	16	1.30
3	Seth I	15	1.22
4	Gupta R	10	0.81
5	Mondal H	10	0.81
6	Rozen WM	10	0.81
7	Wu HY	10	0.81
8	Cheungpasitporn W	8	0.65
9	Egro FM	8	0.65
10	Klang E	8	0.65

Moreover, Table 5 provides a detailed overview of several journals along with their corresponding records, expressed as a percentage of the total, and their respective impact factors (IF) for the year 2023. “Cureus Journal of Medical Science” leads with 127 records, constituting 10.30% of the total publications, and an impact factor of 1.2. Following closely is the “Annals of Biomedical Engineering” with 48 records (3.89%) and an IF of 3.8. “JMIR Medical Education” and “Journal of Medical Internet Research” contribute significantly with 31 records (2.51%) and 20 records (1.62%) respectively, with the latter boasting the highest impact factor among the listed journals at 7.4. Notably, “International Journal of Surgery” stands out with 11 records (0.89%) and an impressive impact factor of 15.3, underscoring its significance in the field. Additionally, “Medical Teacher,” “European Archives of Oto-Rhino-Laryngology,” “Aesthetic Surgery Journal,” “Aesthetic Plastic Surgery,” and “Healthcare” also make valuable contributions to medical literature, each with varying records and impact factors (see Table 6).

**Table 5:** The top 10 most productive journals related to ChatGPT in medical research.

Rank	Journal	Records	Percentage (*N*/1,239)	Impact factor (IF, 2023)
1	Cureus Journal of Medical Science	127	10.30	1.2
2	Annals of Biomedical Engineering	48	3.89	3.8
3	JMIR Medical Education	31	2.51	3.6
4	Journal of Medical Internet Research	20	1.62	7.4
5	Aesthetic Surgery Journal	16	1.30	2.9
6	Medical Teacher	16	1.30	4.7
7	European Archives of Oto-Rhino-Laryngology	15	1.22	2.6
8	Aesthetic Plastic Surgery	13	1.05	2.4
9	International Journal of Surgery	11	0.89	15.3
10	Healthcare	9	0.73	2.8

**Table 6:** The top 10 literatures with the most citations in the field of ChatGPT in medical research.

Rank	Title	Document type	Journal	IF	Total citations
1	ChatGPT utility in healthcare education, research, and practice: systematic review on the promising perspectives and valid concerns	Review	Healthcare	2.8	302
2	Comparing physician and artificial intelligence chatbot responses to patient questions posted to a public social media forum	Article	JAMA Internal Medicine	39.0	204
3	Artificial hallucinations in ChatGPT: implications in scientific writing	Article	Cureus Journal of Medical Science	1.2	186
4	ChatGPT and the future of medical writing	Editorial Material	Radiology	19.0	146
5	Evaluating the feasibility of ChatGPT in healthcare: an analysis of multiple clinical and research scenarios	Article	Journal of Medical Systems	5.3	128
6	Can artificial intelligence help for scientific writing?	Article	Critical Care	15.1	125
7	Generating scholarly content with ChatGPT: ethical challenges for medical publishing	Editorial Material	Lancet Digital Health	30.8	118
8	What if the devil is my guardian angel: ChatGPT as a case study of using chatbots in education	Article	Smart Learning Environments	4.8	114
9	ChatGPT—reshaping medical education and clinical management	Article	Pakistan Journal of Medical Sciences	2.2	104
10	Nonhuman “authors” and implications for the integrity of scientific publication and medical knowledge	Editorial Material	JAMA-Journal of the American Medical Association	120.7	102

### 3.4 Bibliographic coupling analysis of country/region, institution, journal, and author

**Figure 4:**
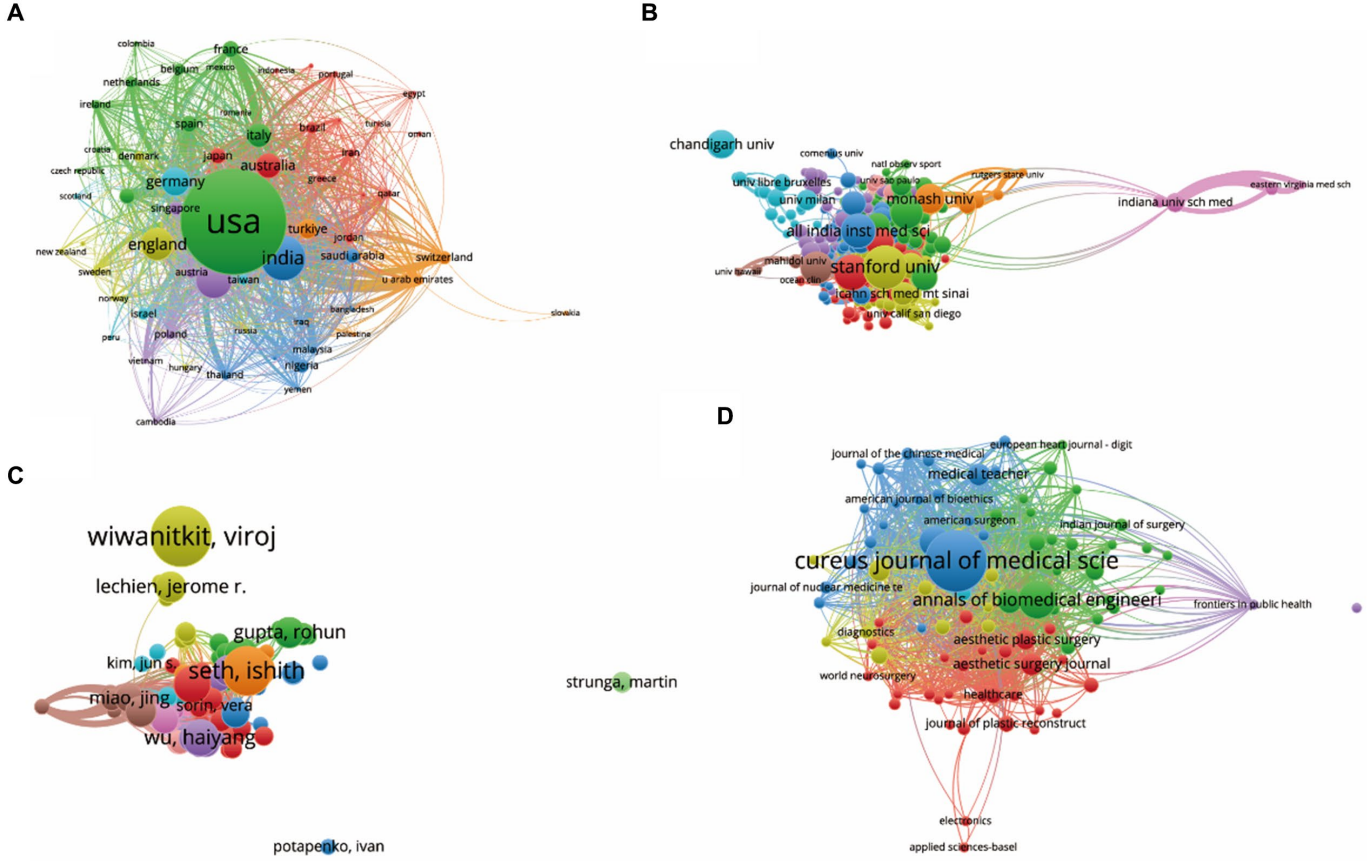
Mapping of bibliographic coupling analysis on medical research revolving around ChatGPT. **(A)** Visualization of the 63 countries/regions contributing to ChatGPT research in the medical domain. **(B)** Cartography of the 277 institutions engaged in ChatGPT research in the medical field. **(C)** Illustration of the 88 identified journals publishing research on ChatGPT in the medical domain. **(D)** Depiction of the 145 authors actively contributing to ChatGPT research in the medical field. Lines connecting different points denote the establishment of a similarity relationship between journals, institutions, countries, or authors. The thickness of the line indicates the strength of the relationship, with thicker lines representing closer links between the entities.

Publications (defined as the minimum number of documents of a country more than 3) originating from 63 countries/regions were analyzed using VOSviewer (Figure 4A). The top five countries with the largest total link strength were as follows: USA (documents = 458, citations = 2,461, total link strength = 148,435), India (documents = 141, citations = 528, total link strength = 51,016), China (documents = 106, citations = 430, total link strength = 49,745), England (documents = 90, citations = 559, total link strength = 42,204), and Canada (documents = 50, citations = 257, total link strength = 32,509).

Papers (defined as the minimum number of documents of an organization that were used more than 3 and the maximum number of organizations per document no more than 25) from 277 institutions were identified and analyzed using VOSviewer (Figure 4B). The top five institutions with the largest total link strength were: Stanford University (documents = 29, citations = 160, total link strength = 19,693), University of Michigan (documents = 14, citations = 67, total link strength = 10,458), National University of Singapore (documents = 14, citations = 38, total link strength = 9,684), University of Toronto (documents = 16, citations = 142, total link strength = 9,490), and University of Jordan (documents = 9, citations = 317, total link strength = 9,168).

Bibliographic coupling was employed to analyze the similarity relationship between documents. VOSviewer identified 89 journals with the highest total link strength (Figure 4C). The top five journals were: Cureus Journal of Medical Science (Impact Factor, IF = 1.2, 2023, total link strength = 11,855), JMIR Medical Education (IF = 3.6, 2023, total link strength = 9,253), Annals of Biomedical Engineering (IF = 3.8, 2023, total link strength = 4,093), Journal of Medical Internet Research (IF = 7.4, 2023, total link strength = 3,906), and Healthcare (IF = 2.8, 2023, total link strength = 2,924).

Publications (defined as the minimum number of documents of an author more than 3) from 145 authors were further analyzed using VOSviewer (Figure 4D). The top five productive authors were: Cheungpasitporn, Wisit (documents = 8, citations = 31, total link strength = 4,641), Miao, Jing (documents = 7, citations = 31, total link strength = 4,351), Thongprayoon, Charat (documents = 7, citations = 31, total link strength = 4,351), Suppadungsuk, Supawadee (documents = 5, citations = 21, total link strength = 3,496), and Sallam, Malik (documents = 5, citations = 308, total link strength = 3,250).

### 3.5 Co-authorship analysis of author, institution, and country

**Figure 5:**
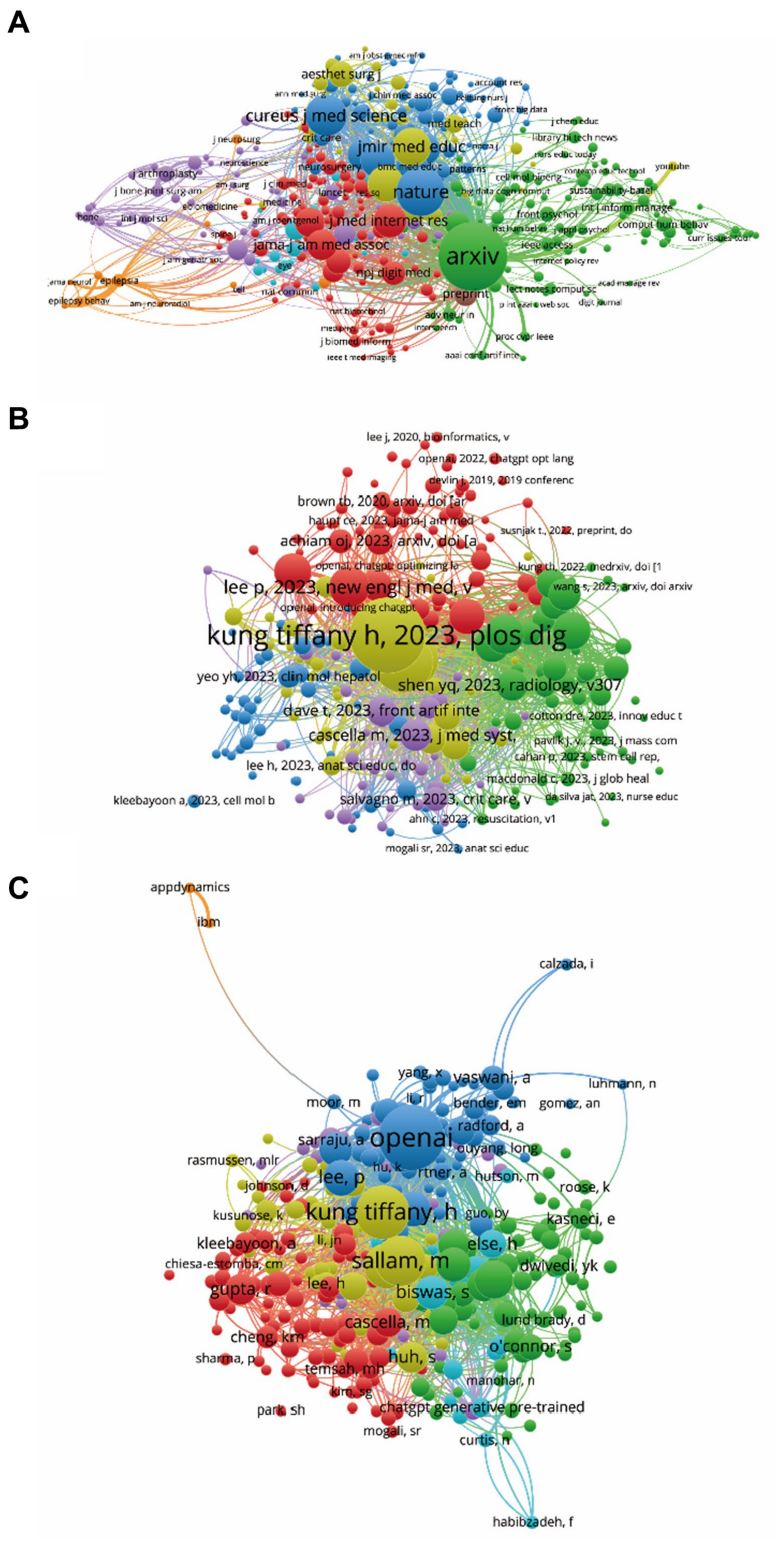
Mapping of co-citation related to ChatGPT in medical research. **(A)** Visualization of co-cited journals pertinent to this field, with 445 distinct journals represented by individual points of varying colors. **(B)** Cartography depicting co-cited references relevant to this field, featuring 247 points in different colors to represent cited references. **(C)** Illustration of co-cited authors within this field, with 316 points of varying colors representing identified authors. The size of the points corresponds to the frequency of citations. Lines connecting different points indicate citation within the same paper, with shorter lines indicating closer connections between two papers. Points of the same color denote affiliation with the same research area.

Co-authorship analysis was conducted to assess the interconnectedness of items based on the total number of coauthored papers. A selection of 63 countries, each with over three papers, underwent analysis using VOSviewer, with results depicted in Figure 5A. The top five countries demonstrating the greatest total link strength were as follows: USA (documents = 458, citations = 2,461, total link strength = 269 times), England (documents = 90, citations = 559, total link strength = 139 times), Italy (documents = 59, citations = 428, total link strength =97 times), India (documents = 141, citations = 528, total link strength = 96 times), and China (documents = 106, citations = 430, total link strength = 95 times).

Furthermore, 247 institutions, each with more than five documents, were subject to analysis via VOSviewer (Figure 5B). The top five institutions exhibiting the greatest total link strength were as follows: Harvard Medical School (documents = 22, citations = 94, total link strength = 62 times), Duke University (documents = 19, citations = 91, total link strength = 46 times), Stanford University (documents = 29, citations = 160, total link strength = 41 times), University of Michigan (documents = 14, citations = 67, total link strength = 41 times), and National University of Singapore (documents = 14, citations = 38, total link strength = 34 times).

Finally, 19 authors, each with over three documents, underwent analysis using VOSviewer, with results displayed in Figure 5C. The top five authors exhibiting the largest total link strength were as follows: Seth, Ishith (documents = 15, citations = 82, total link strength = 37 times), Wu, Haiyang (documents = 10, citations = 74, total link strength =35 times), Cheungpasitporn, Wisit (documents = 8, citations = 31, total link strength = 34 times), Miao, Jing (documents = 7, citations = 31, total link strength =34 times), and Thongprayoon, Charat (documents = 7, citations = 31, total link strength =34 times).

### 3.6 Co-citation analysis of cited journal, reference and author

**Figure 6:**
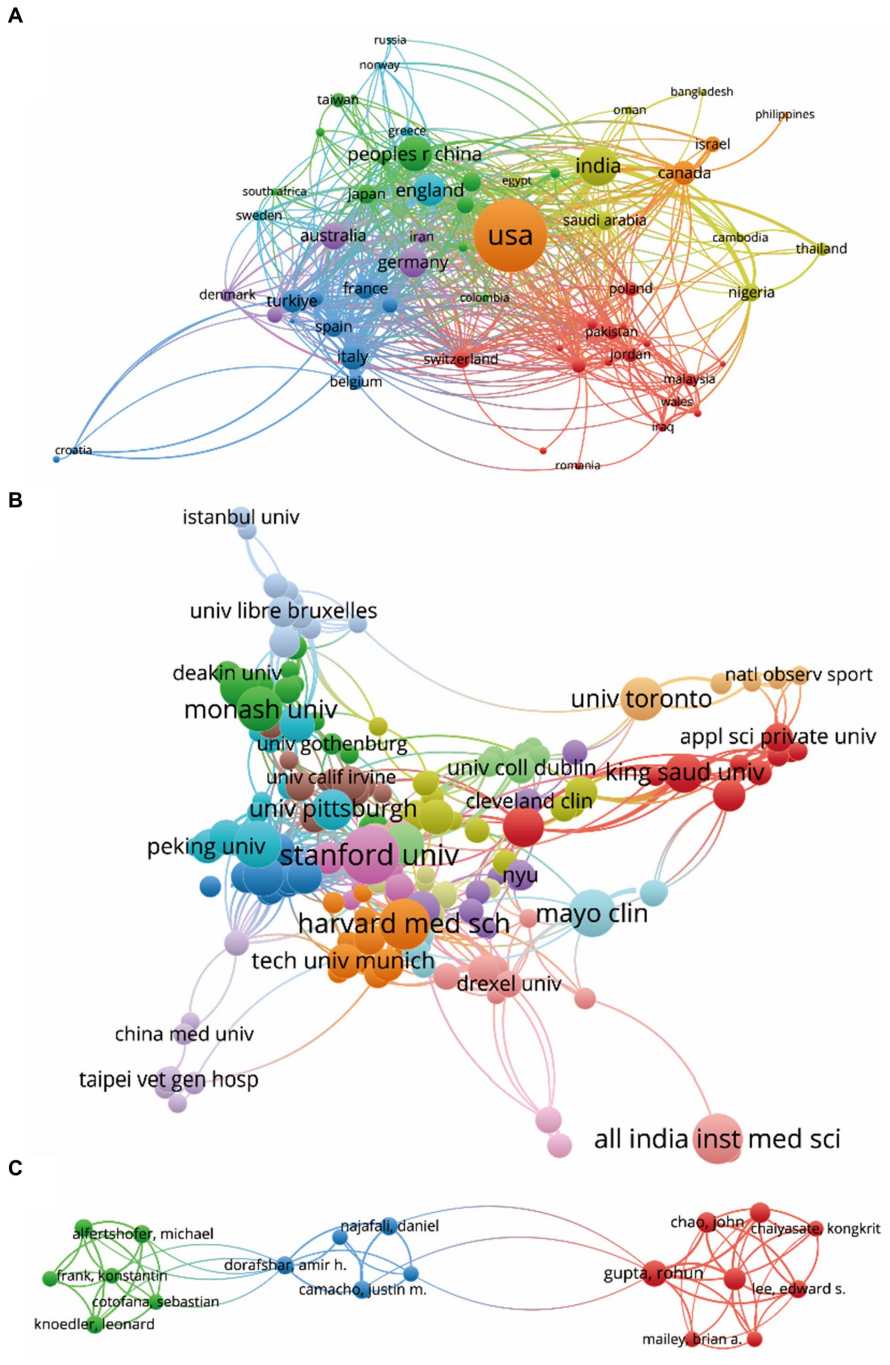
Co-authorship analysis of global research about ChatGPT in medical research. **(A)** Visualization of the co-authorship analysis spanning 63 countries/regions. **(B)** Cartography depicting co-authorship analysis across 247 institutions. **(C)** Illustration of co-authorship analysis involving 19 authors. Lines connecting points signify collaboration between two authors, institutions, or countries, with thickness reflecting the strength of collaboration.

The co-citation analysis aimed to evaluate the relatedness of items based on their co-cited frequencies. Using VOSviewer, the names of journals subjected to co-citation analysis were identified, with only those with citations exceeding 10 considered. As depicted in Figure 6A, a total of 445 journals were analyzed, among which the top five with the highest total link strength were: arXiv (total link strength = 27,195 times), Nature (total link strength = 15,626 times), Cureus Journal of Medical Science (total link strength =14,621 times), medRxiv (total link strength = 10,038 times), and Lancet Digital Health (total link strength = 7,583 times).

Additionally, co-citation analysis was conducted on 247 references via VOSviewer (defined as those cited more than 10 times) (Figure 6B). The top five articles with the greatest total link strength included: Kung Tiffany H, 2023, PLoS Digital Health, 2, e0000198, doi 10.1371/journal.pdig.0000198 (total link strength = 1946 times); Gilson Aidan, 2023, JMIR Medical Education, 9, e45312, doi 10.2196/45312 (total link strength = 1,697 times); Sallam M, 2023, Healthcare-Basel, 11, 887, doi 10.3390/healthcare11060887 (total link strength = 1,369 times); Patel SB, 2023, Lancet Digital Health, 5, e107, doi 10.1016/s2589-7500(23)00021-3 (total link strength =1,051 times); Bockting CL, 2023, Nature, 614, 224, doi 10.1038/d41586-023-00288-7 (total link strength = 1,018 times).

Moreover, a total of 316 authors with a minimum of 10 documents each were analyzed using VOSviewer (Figure 6C). The top five authors with the largest total link strength were identified as OpenAI (total link strength =3,175 times), Sallam, M (total link strength = 2,845 times), Kung Tiffany, H (total link strength =2,533 times), Gilson, Aidan (total link strength =2,201 times), and Huh, S (total link strength =1,438 times).

### 3.7 Co-occurrence analysis of keywords

**Figure 7:**
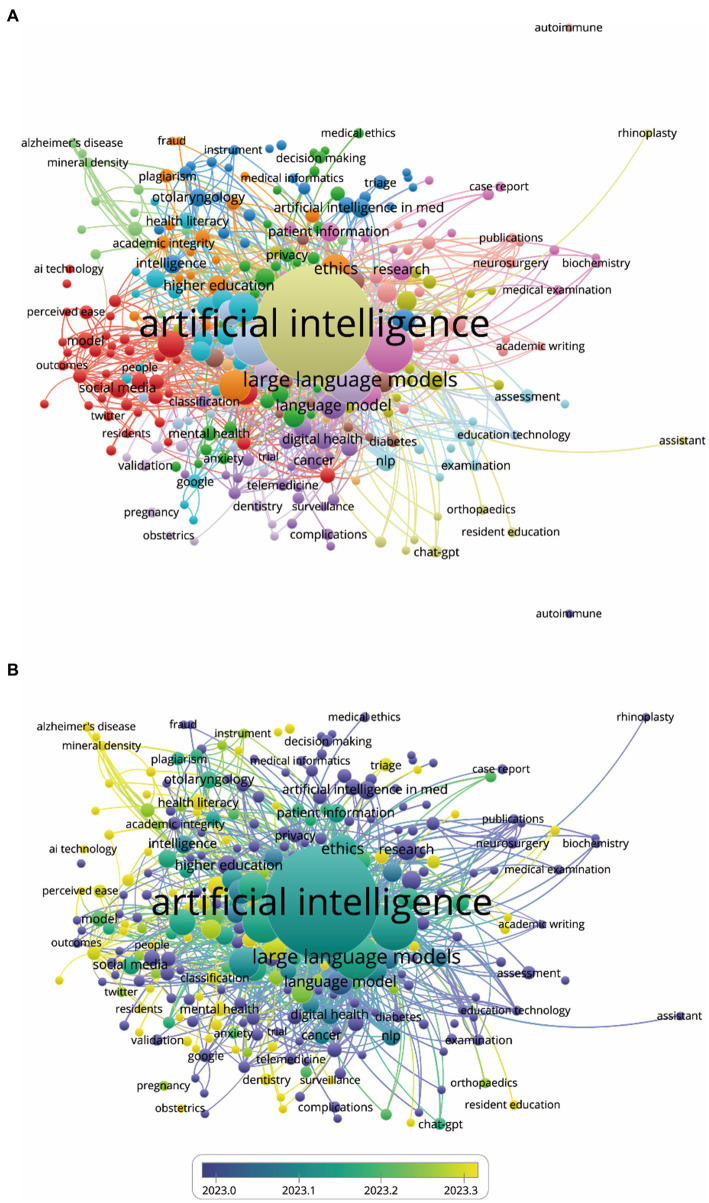
Co-occurrence analysis of global trends and hotspots about ChatGPT in medical research. **(A)** Mapping of keywords in the research on ChatGPT in medical research. **(B)** Distribution of keywords according to the mean frequency of appearance; keywords in yellow appeared later than those in blue.

The objective of co-occurrence analysis is to explore popular research directions and areas, playing a pivotal role in monitoring scientific developments. Keywords, defined as words used at least 3 times in titles/abstracts across all papers, were selected and analyzed using VOSviewer. As shown in Figure 7A, the 313 identified keywords were mainly discovered and the top 10 keywords with highest total link strength were shown as follows: artificial intelligence (occurrences = 513, total link strength = 1,975), ai (occurrences = 101, total link strength = 537), chatbot (occurrences = 81, total link strength = 458), large language models (occurrences = 84, total link strength = 428), medical education (occurrences = 89, total link strength = 416), natural language processing (occurrences = 65, total link strength = 357), machine learning (occurrences = 65, total link strength = 349), artificial intelligence (occurrences = 68, total link strength = 324), large language model (occurrences = 67, total link strength = 280), language model (occurrences = 26, total link strength = 223). Furthermore, keywords were color-coded based on their average appearance in all published papers (Figure 7B), with blue representing earlier appearances and yellow indicating later ones. Notably, the trends across most studies showed minimal variation, suggesting a consistent distribution of research interests across various fields in the future.

**Figure 8:**
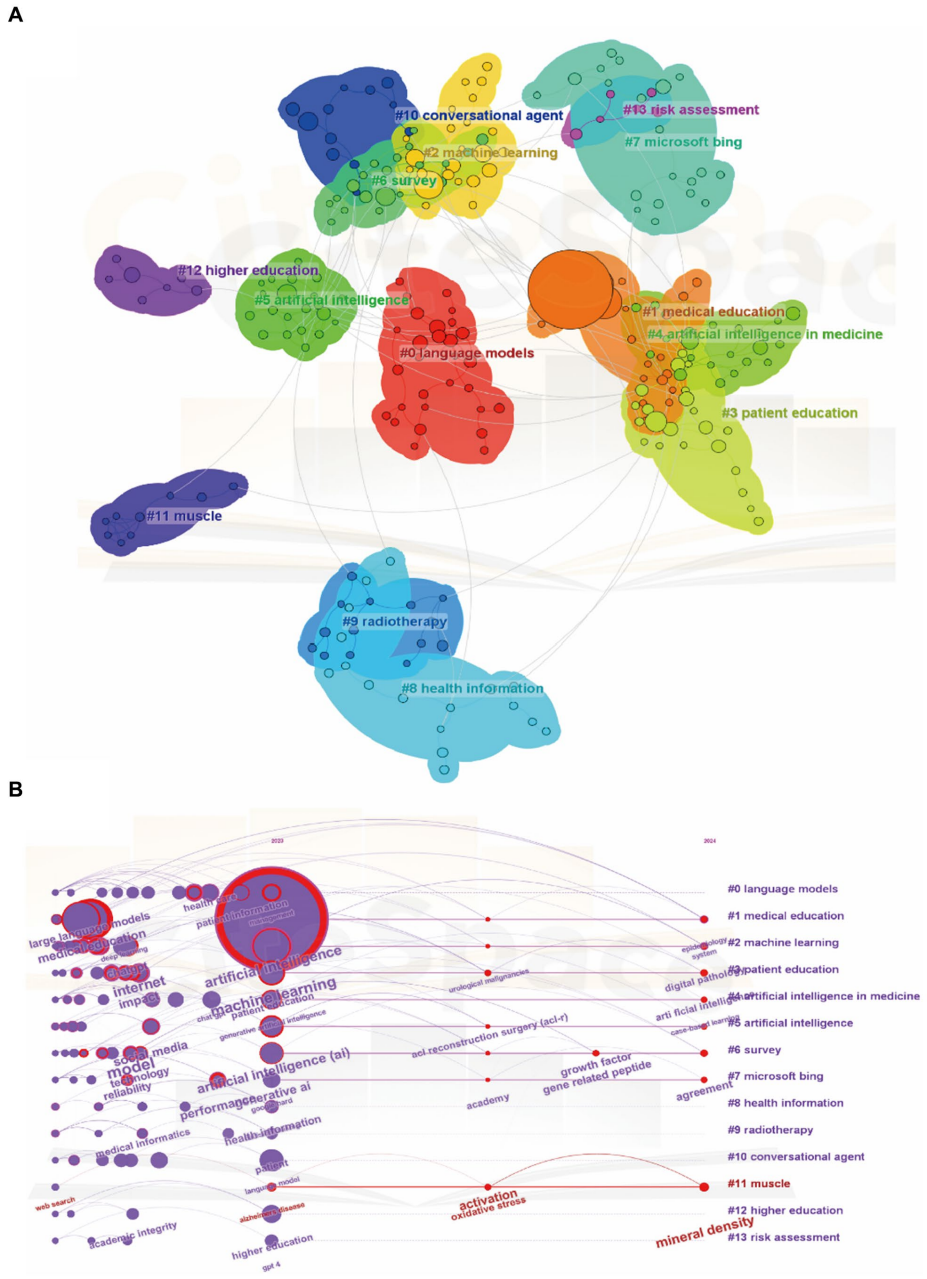
Mapping of keywords in studies concerning ChatGPT in medical research. **(A)** Visualization of keyword clustering from 2023-01-01 to 2024-01-31. **(B)** Visualization of keyword timeline from 2023-01-01 to 2024-01-31.

A network map was constructed to illustrate keyword clusters (Figure 8A), with each node representing a prominent keyword using CiteSpace. The clusters of keywords, in sequential order, include #0 language models, #1 medical education, #2 machine learning, #3 patient education, #4 artificial intelligence in medicine, #5 artificial intelligence, #6 survey, #7 Microsoft Bing, #8 health information, #9 radiotherapy, #10 conversational agent, #11 muscle, #12 higher education, and #13 risk assessment. Additionally, we analyzed the transformation of keywords over the past year and visualized the timeline of related keywords to provide detailed insights.

As Figure 8B indicates, there is a significant variation in the frequency of keywords over time, suggesting a shift in research focus. Within cluster #0, language models, the top five keywords include artificial intelligence, colorectal cancer, surveillance interval, health education, and pediatric palliative critical care. Similarly, within cluster #1, medical education, the top five keywords are artificial intelligence, medical education, macular edema, wet macular degeneration, and dry macular degeneration. Moving to cluster #2, machine learning, the top five keywords comprise artificial intelligence, large language model, generative pre-trained transformer, Google Bard, and sports medicine. Within cluster #3, patient education, key terms include artificial intelligence, neck cancer, common questions, health education, and medical education. Regarding cluster #4, artificial intelligence in medicine, the top five keywords encompass artificial intelligence, medical education, emergency medicine, specialty examination, and overall quality. In cluster #5, artificial intelligence, notable terms include artificial intelligence, natural language processing, patient education materials, health literacy, and anterior cruciate ligament. Moreover, within cluster #6, the survey, significant keywords include artificial intelligence, neural regulation, sympathetic nervous system, fracture healing, and central nervous system. Furthermore, in cluster #7, Microsoft Bing, keywords consist of artificial intelligence, head-neck surgery, surgical training, provisional diagnosis, and medical education. In cluster #8, health information, top terms encompass artificial intelligence, large language model, disruptive technology, health information, and data security. Similarly, cluster #9, radiotherapy, includes keywords such as artificial intelligence, large language model, natural language processing, clinical decision support, and clinical guidelines. Within cluster #10, conversational agent, key terms comprise artificial intelligence, language model, large language models, conversational agent, and conversational agents. Moving to cluster #11, muscle, significant keywords include artificial intelligence, Alzheimer’s disease, fracture healing, COVID-19, and bone disease. In cluster #12, higher education, notable terms include artificial intelligence, academic integrity, language model, tertiary education, and post-digital education. Finally, cluster #13, risk assessment, encompasses artificial intelligence, risk assessment, powered algorithm, refractive surgery, and data analysis.

## 4 Discussion

The sudden rise of large language models (LLMs), particularly OpenAI’s ChatGPT, into public consciousness and their rapid uptake in academia and medicine underscores their versatility, prompting a critical examination of the ethical dilemmas they pose (26). This study conducted a bibliometric and visualization analysis of ChatGPT in medical research from January 1, 2023, to January 31, 2024, leveraging the advancements in bibliometric software. Such analyses are increasingly popular as they offer an intuitive and systematic understanding of the development process and trends in a specific field. Moreover, they aid in identifying new research hotspots and significant milestones, benefiting researchers, particularly beginners, in navigating the field.

### 4.1 Trend of global publications

The analysis presented paints a fascinating picture of the global landscape of ChatGPT research in the medical field. One of the most striking aspects is the exponential growth in the number of publications over just one year, from January 2023 to January 2024. Starting from a modest 26 articles in February 2023, the count surged to over 100 articles by January 2024, indicating a burgeoning interest and engagement in this area of study (Figures 1, 2). This meteoric rise in publications reflects not only the increasing importance of ChatGPT in medical research but also the rapid pace of advancement and innovation within the field (Figure 2A).

Interestingly, while the number of publications has been on the rise, the RRI has shown a relatively stable trend around the baseline level over the same period. This suggests that while the volume of research output is increasing, the overall level of interest and engagement in ChatGPT in medical research has remained consistent (Figure 2A). It would be intriguing to delve deeper into the factors driving this stability in RRI and explore whether it reflects a mature and sustained interest in the field or if there are underlying dynamics at play (27).

Moreover, the global distribution of contributions is noteworthy, with research emanating from a diverse array of countries and regions. While it is not surprising to see the USA spearheading this effort with the largest share of publications, it’s encouraging to observe significant contributions coming from countries like India, China, and England (28). This highlights the truly global nature of ChatGPT research in the medical domain and underscores the importance of international collaboration and cooperation in advancing scientific knowledge and innovation.

### 4.2 Quality and status of global publications

Moving on to the quality of publications, citation frequencies serve as a valuable metric for assessing the impact and influence of research outputs. The fact that publications from the USA have garnered the highest total citation frequencies underscores the significant impact of research originating from this region (Figure 3A). However, it’s important to recognize the contributions of other countries like England, India, Australia, and China, which have also secured notable citation frequencies, indicating the global relevance and impact of their research endeavors (Figure 3A). The analysis of average citation frequencies offers further insights into the quality of publications from different countries and regions. It is intriguing to see countries like Australia and Italy leading in this regard, highlighting the high impact and citation rates of research outputs originating from these regions (Figure 3B). This underscores the importance of not only producing a high volume of research but also ensuring its quality and impact. Additionally, the *H*-index provides a comprehensive measure of both the quantity and citation impact of research outputs, offering a more nuanced understanding of the influence of different countries and regions in the field of ChatGPT in medical research (Figure 3C) (29). Once again, the USA emerges as a leader in this aspect, boasting the highest *H*-index, followed closely by countries like Australia, England, India, and China. This reaffirms the influential role played by these countries in shaping the discourse and direction of research in this dynamic and rapidly evolving field (30).

Furthermore, the comprehensive analysis presented underscores the dynamic and multifaceted nature of ChatGPT research in the medical domain, highlighting the contributions of institutions, funding sources, researchers, and journals in advancing knowledge and driving innovation in this exciting field. In examining the top institutions by publication volume, it’s evident that the USA dominates the landscape, with Harvard University, Stanford University, and the University of California System consistently occupying the top positions (Figure 2C and Table 1). This concentration of publications in a handful of prestigious institutions underscores the significant role played by these academic powerhouses in driving forward the frontiers of ChatGPT research. However, it’s also noteworthy that institutions like the University of London and the National University of Singapore manage to secure notable positions within the top ten, highlighting the global reach and diversity of contributors in this field (Table 1). Turning to funding sources, it’s interesting to observe the dominance of American institutions, with the National Institutes of Health (NIH) and the United States Department of Health and Human Services leading the pack (Table 2). This underscores the critical role of government funding in supporting research endeavors in the medical domain, particularly in cutting-edge areas like ChatGPT. The presence of the National Natural Science Foundation of China (NSFC) among the top funding sources also reflects China’s growing investment and interest in advancing research in this field (Table 2). In exploring research orientations, it is fascinating to see the diverse array of topics covered, ranging from General Internal Medicine and Surgery to education, healthcare sciences, and engineering (Table 3). This multifaceted approach reflects the interdisciplinary nature of ChatGPT research and its applications across various domains within medicine. Additionally, the significant contributions from fields like computer science, radiology, medical informatics, orthopedics, and oncology highlight the breadth and depth of research being conducted in these specialized areas. Delving into the prolific authors, it is impressive to see individuals like Wiwanitkit V, Kleebayoon A, and Seth I leading the pack with a substantial number of publications to their names (Table 4). Their contributions not only underscore their expertise and dedication to advancing knowledge in ChatGPT research but also serve as inspiration for aspiring researchers in the field. Lastly, the analysis of journals provides valuable insights into the dissemination of research findings, with journals like “*Cureus Journal of Medical Science*” and “*Annals of Biomedical Engineering*” emerging as prominent platforms for publishing ChatGPT research (Table 5). The varying impact factors of these journals reflect their respective influence and prestige within the academic community, with journals like “*Journal of Medical Internet Research*” boasting particularly high impact factors, indicative of their significant contribution to shaping the discourse in the field.

### 4.3 Bibliometrics and visual analysis

The comprehensive analysis provided offers valuable insights into the complex network of relationships within the ChatGPT research landscape. It underscores the collaborative and interdisciplinary nature of research in this field and highlights key players driving innovation and advancement in the medical domain (31). The bibliographic coupling analysis offers valuable insights into the interconnectedness of publications, institutions, journals, and authors in the realm of ChatGPT research within the medical domain. Examining the contributions of different countries/regions, it’s clear that the USA leads the pack, followed closely by India, China, England, and Canada. This underscores the global reach and collaboration driving advancements in this field (24). Institutions also play a pivotal role, with Stanford University, University of Michigan, National University of Singapore, University of Toronto, and University of Jordan emerging as key contributors (Figure 4). Their research output and citation impact highlight their significance in shaping the discourse and direction of ChatGPT research.

Journals serve as crucial platforms for disseminating research findings, with *Cureus Journal of Medical Science, JMIR Medical Education, Annals of Biomedical Engineering, Journal of Medical Internet Research*, and *Healthcare* emerging as prominent venues for publishing ChatGPT research (Figure 4C). Their impact factors and total link strength underscore their influence in the academic community. Analyzing authors provides insights into individual contributions, with Cheungpasitporn, Wisit; Miao, Jing; Thongprayoon, Charat; Suppadungsuk, Supawadee; and Sallam, Malik emerging as prolific contributors to the field (Figure 5C). Their research output and citation impact reflect their expertise and dedication to advancing knowledge in ChatGPT research.

Furthermore, co-authorship analysis sheds light on collaborative networks among countries, institutions, and authors (Figure 5). The interconnectedness of items highlights the importance of international collaboration in driving innovation and progress in ChatGPT research. Co-citation analysis delves into the relatedness of journals, references, and authors based on their co-cited frequencies. Journals like *arXiv, Nature, Cureus Journal of Medical Science, medRxiv,* and *Lancet Digital Health* emerge as central to the discourse, indicating their significance in shaping research directions and trends.

### 4.4 Analysis of research hotspots

The co-occurrence analysis conducted in this study provides a comprehensive overview of research themes and trends within the domain of ChatGPT in Medical Research (32). Leveraging keywords extracted from titles and abstracts, the study aimed to delineate prominent themes and trends shaping the field, revealing a rich landscape of topics that reflects the multifaceted nature of research in this domain. By identifying prominent keywords, thematic clusters, and temporal variations, the analysis offers valuable insights for researchers, practitioners, and policymakers alike, facilitating informed decision-making and driving future research agendas in this rapidly evolving field (33).

The identification of 313 keywords within the realm of ChatGPT research signifies the expansive breadth and depth of scholarly interests in this domain. By focusing on keywords that occur at least three times across all papers, the study underscores the richness of research topics and the multifaceted nature of investigations surrounding ChatGPT. Among these keywords, prominent themes such as artificial intelligence, AI, chatbot, large language models, medical education, natural language processing, and machine learning have emerged with the highest total link strength (Figures 7, 8). The prominence of these keywords suggests a strong emphasis on the application of advanced AI techniques within the context of medical education and healthcare settings (34, 35). This observation reflects the growing recognition of the potential of ChatGPT and similar technologies to revolutionize medical education, enhance healthcare delivery, and improve patient outcomes (36, 37). Specifically, the integration of AI-driven solutions in medical education holds promise for facilitating personalized learning experiences, improving diagnostic accuracy, and optimizing treatment strategies (38). Moreover, the utilization of large language models and natural language processing techniques enables healthcare providers to extract valuable insights from vast amounts of textual data, leading to more informed decision-making and enhanced patient care (39).

Furthermore, the uniform color-coding of keywords based on their average appearance in published papers provides valuable insights into the temporal dynamics of research interests within the ChatGPT landscape. Despite the evolving nature of research trends, the consistent distribution of keywords over time indicates the presence of enduring areas of focus amidst changing paradigms (Figure 8). This suggests a sustained interest in core themes such as AI, machine learning, and natural language processing, which continue to drive innovation and shape the trajectory of research within the field. Additionally, the comprehensive analysis of keywords underscores the dynamism and vibrancy of research surrounding ChatGPT. By elucidating prominent themes and trends, this research provides valuable guidance for scholars, practitioners, and policymakers seeking to navigate the complex landscape of AI-driven healthcare innovation (35). Moreover, it highlights the ongoing evolution of ChatGPT research and its potential to revolutionize medical education and healthcare delivery in the years to come (40, 41).

The utilization of CiteSpace to construct a network map has greatly enhanced our understanding of the intricate web of research themes within the ChatGPT landscape (42). By visually representing keyword clusters, this approach has provided researchers with a powerful tool to navigate the complex research terrain and identify key areas of interest. Each node in the network map represents a prominent keyword, with the connections between nodes offering insights into the interconnectedness and co-occurrence patterns of research themes. The sequential order of clusters, spanning from language models and medical education to patient education and artificial intelligence in medicine, highlights the diverse yet interconnected nature of research endeavors within the field of ChatGPT (43). This sequential arrangement underscores the multidimensional nature of research in this domain, with distinct thematic groupings reflecting the multifaceted applications and implications of ChatGPT technology. From enhancing language models to revolutionizing medical education and healthcare delivery, the research landscape surrounding ChatGPT encompasses a wide range of disciplines and specialties (44, 45).

Moreover, the analysis of keyword transformations over time has provided researchers with a nuanced understanding of shifting research priorities and emerging areas of interest. By tracking the evolution of keywords across different clusters, researchers can identify emerging trends, anticipate future research directions, and adapt their research agendas accordingly (46). This dynamic approach to keyword analysis enables researchers to stay abreast of the latest developments in the field and harness the full potential of ChatGPT technology in addressing pressing societal challenges. In addition, the construction of a network map using CiteSpace has been instrumental in facilitating the visualization of keyword clusters and revealing the underlying structure of the research landscape (42). By uncovering distinct thematic groupings and tracking keyword transformations over time, this approach has provided valuable insights into the evolving nature of research within the field of ChatGPT. Moving forward, researchers can leverage these insights to drive innovation, foster interdisciplinary collaboration, and advance our understanding of ChatGPT technology and its applications in various domains.

The dynamic analysis of keyword frequency over time, depicted in Figure 8B, provides valuable insights into the evolving research landscape and shifting priorities within specific thematic clusters. This temporal analysis reveals fluctuations in keyword frequency, reflecting the dynamic nature of research trends and the emergence of new areas of interest within the field of ChatGPT. Within the language models cluster, keywords such as artificial intelligence, colorectal cancer, and health education exhibit variations in frequency over time (47, 48). These fluctuations suggest a dynamic research landscape characterized by evolving priorities and emerging subtopics. For example, the increased frequency of keywords related to colorectal cancer may indicate a growing emphasis on the application of language models in oncology research (49, 50), while fluctuations in health education-related keywords may reflect changing perspectives on the role of ChatGPT in patient education and healthcare communication (51). Similarly, in clusters focusing on medical education, machine learning, patient education, and artificial intelligence in medicine, variations in keyword frequency underscore evolving research trends and emerging areas of interest (52). For instance, the integration of AI technologies in medical education and healthcare delivery is becoming increasingly prominent, as evidenced by the growing frequency of keywords related to artificial intelligence and medical education (53, 54). This suggests a shift towards the adoption of ChatGPT and similar technologies to enhance learning experiences, improve diagnostic accuracy, and optimize treatment strategies within the medical domain.

According to CiteSpace analysis, the 14 clusters of keywords can be categorized into four thematic groups: Artificial Intelligence (AI) and Machine Learning (ML), Education and Training, Healthcare Applications, and Data Analysis and Technology. The first group includes points related to the application of AI and ML in medicine and conversational agents. The application of AI and ML in medicine has revolutionized healthcare delivery by enhancing diagnostic accuracy, treatment planning, and patient care (55, 56). AI-powered systems can analyze vast amounts of medical data to identify patterns, predict diseases, and recommend personalized treatment plans, leading to more efficient and effective healthcare outcomes (57). Additionally, conversational agents, powered by AI, facilitate patient-provider communication, appointment scheduling, and health monitoring, improving accessibility and patient engagement (58). In conclusion, the integration of AI and ML technologies in medicine holds immense potential to transform healthcare delivery, making it more personalized, efficient, and patient-centered.

Education and Training encompass medical and higher education, along with patient education. Education and Training play a crucial role in the adoption and implementation of AI and ML technologies in healthcare. In medical education, incorporating AI and ML concepts into curricula prepares future healthcare professionals to leverage these technologies effectively in clinical practice, research, and decision-making processes (38). Similarly, in higher education, specialized programs and courses focusing on AI and ML equip students with the skills and knowledge needed to innovate and advance in the field of healthcare technology (36). Additionally, patient education initiatives aimed at raising awareness about AI-driven healthcare solutions empower individuals to make informed decisions about their health and participate actively in their care (59). Together, these educational efforts foster a workforce and patient population that is knowledgeable, skilled, and prepared to harness the benefits of AI and ML in healthcare.

Healthcare Applications involve language models, radiotherapy, health information, and muscle-related research. Healthcare Applications of AI and ML technologies encompass a diverse range of areas crucial to improving patient care and outcomes. Language models, powered by AI, aid in natural language processing tasks, enabling more accurate and efficient documentation, transcription, and analysis of medical records and patient interactions (60). In radiotherapy, AI algorithms enhance treatment planning and delivery, optimizing radiation doses to target tumors while minimizing damage to healthy tissues (61). Moreover, AI-driven health information systems facilitate data management, analysis, and sharing, promoting seamless collaboration among healthcare providers and enhancing clinical decision-making processes (62). Muscle-related research benefits from AI and ML applications in biomechanical modeling, rehabilitation, and performance optimization, offering insights into musculoskeletal health and injury prevention (63). Therefore, these healthcare applications highlight the transformative potential of AI and ML in advancing diagnosis, treatment, and overall patient care across various medical domains.

Moreover, Data Analysis and Technology, encompassing survey methodologies, Microsoft Bing, and risk assessment, showcase the multifaceted utilization of AI and ML across different domains. Survey methodologies benefit from AI-driven data analysis techniques, enabling researchers to gather, process, and interpret survey data more efficiently and accurately (64). Microsoft Bing leverages AI algorithms to enhance search engine functionality, providing users with more relevant and personalized search results based on their preferences and behavior (65). In risk assessment, AI and ML technologies empower organizations to analyze vast amounts of data and identify potential risks, enabling proactive measures to mitigate and manage these risks effectively (66). These examples underscore the versatility and transformative potential of AI and ML in optimizing processes, enhancing decision-making, and driving innovation across various sectors, from research and technology to business and risk management.

### 4.5 Limitations

Several limitations warrant discussion: due to our bibliometric software’s constraints, publication bias may arise from excluded databases, necessitating the inclusion of additional sources and more robust software in future studies. Additionally, the study’s focus on English articles may introduce omissions, warranting consideration of non-English language literature in future investigations. Moreover, neglecting temporal data visualization may introduce prediction bias in identifying research hotspots, underscoring the importance of incorporating timeline-specific keywords for a more nuanced analysis. Furthermore, daily updates may overlook influential new studies, emphasizing the need for continuous monitoring to capture emerging research. Consultation with experts was crucial for resolving encountered issues during data selection, ensuring a rigorous approach despite the involvement of only two authors. While data were meticulously cleaned and analyzed by coauthors, potential inaccuracies persist, highlighting the inherent limitations of this approach. Expanding to include databases like Scopus could enhance comprehensiveness and representativeness, aligning with standard bibliometric practices.

## 5 Conclusion

In summary, this study provides a comprehensive bibliometric and visualization analysis aimed at evaluating global trends in the integration of ChatGPT within medical research from January 1, 2023, to January 31, 2024. Prominent contributors to this field include the USA, Harvard University, and the National Institutes of Health (NIH). The *Cureus Journal of Medical Science* emerged as the most prolific publisher of research on this topic. Furthermore, our findings highlight key clusters of keywords such as “Artificial Intelligence (AI) and Machine Learning (ML),” “Education and Training,” “Healthcare Applications,” and “Data Analysis and Technology,” which likely signify current research hotspots and emerging frontiers in the field.

## Data availability statement

The original contributions presented in the study are included in the article/supplementary material, further inquiries can be directed to the corresponding author.

## Author contributions

LL: Conceptualization, Data curation, Formal analysis, Methodology, Resources, Software, Validation, Visualization, Writing – original draft. SQ: Conceptualization, Data curation, Formal analysis, Investigation, Methodology, Writing – original draft. HZ: Conceptualization, Data curation, Formal analysis, Investigation, Methodology, Writing – original draft, Writing – review & editing. LK: Formal analysis, Investigation, Methodology, Project administration, Writing – original draft. ZX: Formal analysis, Investigation, Methodology, Supervision, Writing – review & editing. ZJ: Methodology, Supervision, Validation, Writing – review & editing. PZ: Conceptualization, Funding acquisition, Investigation, Project administration, Supervision, Validation, Visualization, Writing – review & editing.
